# Bilateral inverted and impacted maxillary third molars: A case report

**DOI:** 10.4317/jced.52389

**Published:** 2015-07-01

**Authors:** Nedal Abu-Mostafa, Ali Barakat, Tareq Al-Turkmani, Abdulaziz Al-Yousef

**Affiliations:** 1 BDS. MSc. Lecturer in Oral and Maxillofacial Surgery, Riyadh Colleges of Dentistry and Pharmacy, Oral and Maxillofacial Surgery and Diagnostic Science Department, Dental Hospital (Munessya) Riyadh, Kingdom of Saudi Arabia; 2 BDS. Dental Intern, Riyadh Colleges of Dentistry and Pharmacy, Dental Hospital (Munessya) Riyadh, Kingdom of Saudi Arabia; 3 Dental student, Riyadh Colleges of Dentistry and Pharmacy, Dental Hospital (Munessya) Riyadh, Kingdom of Saudi Arabia

## Abstract

Bilateral inverted third molar impaction is an extremely rare condition. We reported the case of a 50-year-old female patient with bilateral inverted and impacted maxillary third molars. Both were asymptomatic and pathology free clinically and radiographically. Surgical extraction of these inverted third molars with inaccessible positions requires an aggressive bone removal on the tuberosity bilaterally. Moreover, it contains a high risk of displacement of the inverted third molar into the maxillary sinus. Conservative management was the choice, with the patient’s agreement, and the inverted third molars were left in situ.

** Key words:**Bilateral inverted, maxillary third molar, upper impacted tooth.

## Introduction

An impacted tooth has failed to fully erupt to the assumed normal functional position in the occlusal plane within the expected time (1). Prevention of eruption in the dental arch may be caused by a lack of adequate space in the arch, obstruction in the eruptive pathway of the tooth (2) soft tissue, or bony lesions, malposition of the tooth germ, or a genetic component (3).

A review of the literature showed that the most frequently impacted tooth was the mandibular third molar, followed by the maxillary third molar, maxillary canine, and mandibular premolar (1,2,4).

According to Winter’s classification, patterns of angulation for third molar impaction are vertical, mesioangular, horizontal, distoangular, buccolingual, and inverted (5). An inverted upper tooth has the crown pointing upward while the root apex points toward the alveolar crest (6). Inversion may develop because of atypical proliferation of odontogenic epithelium before development of the tooth germ (7).

The aim of the current report is to present a very rare case of bilateral inverted and impacted maxillary third molars which was incidentally detected.

## Case Report

**Figure 1:**
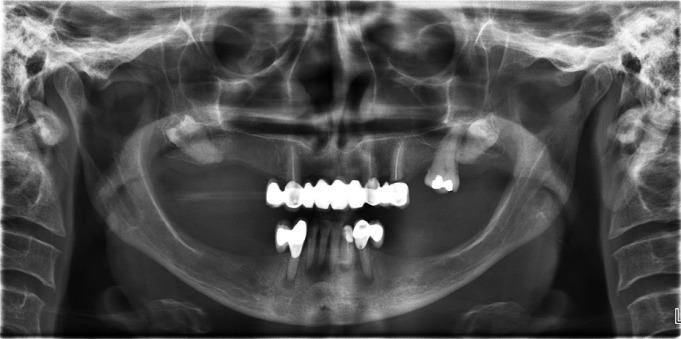
Bilateral inverted maxillary third molars.

**Figure 2:**
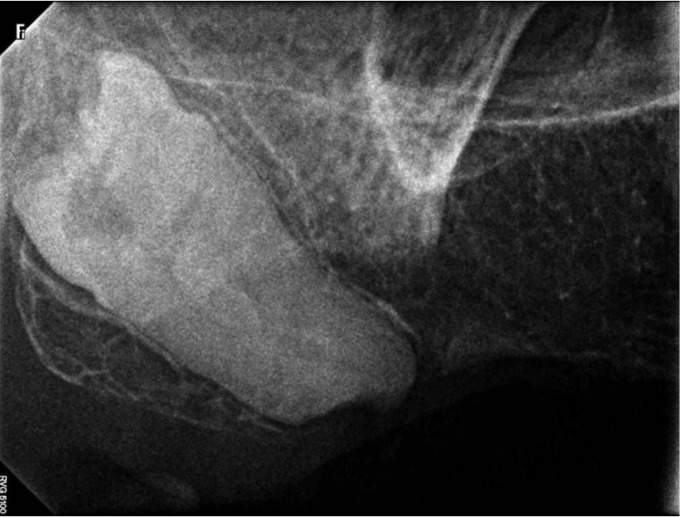
The right inverted upper third molar.

**Figure 3:**
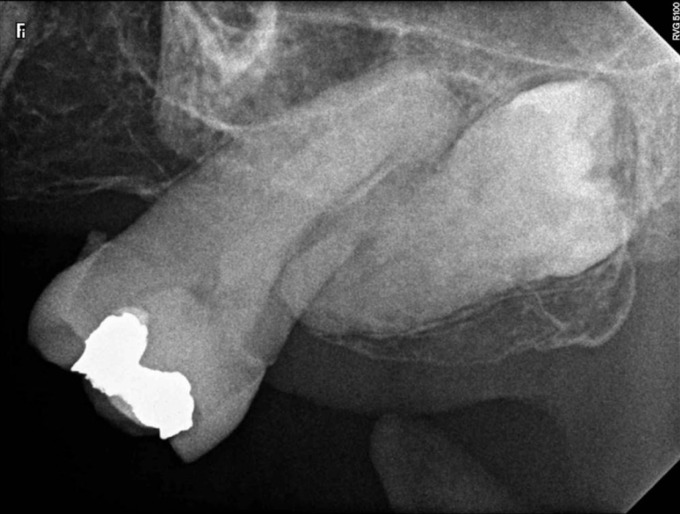
The left inverted upper third molar.

A 50-year-old female patient presented to the institute’s dental hospital complaining of pain and mobility in the lower anterior teeth. The patient’s medical history included a resected malignant tumor in the intestine followed by chemotherapy, which had ended one year ago. Intraoral examination revealed mobility and deep caries on the lower incisors, defective restorations on the lower canines, and bilateral missing lower posterior teeth. In the maxilla, there was a defective upper fixed partial denture exten-ding from the right canine to the left second premolar in addition to a missing right first molar, left first molar, and left second molar. A dental panoramic radiograph and peri-apical films showed multiple recurrent caries on the abutments of the restorations, bone resorption, and peri-apical radiolucencies on the lower anterior teeth. The most interesting aspect was the presence of bilateral impacted maxillary third molars, which were inverted and distally directed (Fig. 1). Both were asymptomatic and pathology free clinically and radiographically. The crown of the right inverted third molar was very close to the maxillary sinus (Fig. 2). On the left side, the upper second molar existed between the inverted third molar and the sinus (Fig. 3).

The patient was informed about the presence of the impacted and inverted maxillary third molars. Then the case was discussed with the prostodontist and the final treatment plan was to create a removable complete denture after clearance of the available teeth. Regarding the inverted maxillary third molars, treatment options discussed were to extract or to leave in place. Thereafter, the treatment options were fully explained to the patient, including the risks versus the benefits of surgical removal of such impacted teeth. Finally, conservative management was selected, with the patient’s agreement, and the inverted third molars were left in situ. This case report was registered in the institute research center with a registration number of (FUGRP/2014/175).

## Discussion

Only a few cases of inverted and impacted third molars have been reported in the literature (7-10). However, inversions of other teeth have been reported, including the lower second premolar (11), upper second premolar (12), upper primary central incisors (3), lower primary incisors (13), and supernumerary tooth in anterior maxilla (9).

No definitive treatment protocols exist for the removal of inverted teeth. The safest protocol is conservative treatment (7,12) in which the teeth are not extracted until they produce pathological signs. However, the patient should be subjected to periodic clinical and radiological evaluation to detect such alterations as quickly as possible (14). Furthermore, the patient should be aware of the indications, contraindications, risks, and benefits of conservative management and surgical removal of impacted teeth and participate in the management decision (6,14).

In this study, conservative treatment was the choice for the bilateral inverted upper third molars because they were pathology free and fully covered by bone and mucosa, which constitute effective barriers against bacterial invasion (15). Additionally, we considered the medical condition and age of the patient, as well as the anticipated local complications associated with removal of the teeth. Surgical extraction of inverted third molars with inapproachable positions requires an aggressive bone removal on the tuberosity bilaterally, which is a major disadvantage since it will negatively affect denture stability and retention. Moreover, it contains a high risk of displacement of the inverted third molar into the maxillary sinus.

To the best of our knowledge, no reported case of bilateral inverted and impacted upper third molar exists in PubMed or Google Scholar database. This study presented an extremely rare occurrence of bilateral inverted and impacted third molars in the maxilla which were subjected to conservative treatment.
